# Nanocontact Disorder in Nanoelectronics for Modulation of Light and Gas Sensitivities

**DOI:** 10.1038/srep13035

**Published:** 2015-08-11

**Authors:** Yen-Fu Lin, Chia-Hung Chang, Tsu-Chang Hung, Wen-Bin Jian, Kazuhito Tsukagoshi, Yue-Han Wu, Li Chang, Zhaoping Liu, Jiye Fang

**Affiliations:** 1 Department of Physics, National Chung Hsing University, Taichung, 40227, Taiwan; 2 Department of Electrophysics, National Chiao Tung University, Hsinchu 30010, Taiwan; 3 WPI Center for Materials Nanoarchitechtonics (WPI-MANA), National Institute for Materials Science (NIMS), Tsukuba, Ibaraki 305-0044, Japan; 4 Department of Materials Science, National Chiao Tung University, Hsinchu 30010, Taiwan; 5 Department of Chemistry, State University of New York at Binghamton, Binghamton, New York 13902-6000, USA

## Abstract

To fabricate reliable nanoelectronics, whether by top-down or bottom-up processes, it is necessary to study the electrical properties of nanocontacts. The effect of nanocontact disorder on device properties has been discussed but not quantitatively studied. Here, by carefully analyzing the temperature dependence of device electrical characteristics and by inspecting them with a microscope, we investigated the Schottky contact and Mott’s variable-range-hopping resistances connected in parallel in the nanocontact. To interpret these parallel resistances, we proposed a model of Ti/TiO_*x*_ in the interface between the metal electrodes and nanowires. The hopping resistance as well as the nanocontact disorder dominated the total device resistance for high-resistance devices, especially at low temperatures. Furthermore, we introduced nanocontact disorder to modulate the light and gas responsivities of the device; unexpectedly, it multiplied the sensitivities compared with the intrinsic sensitivity of the nanowires. Our results improve the collective understanding of electrical contacts to low-dimensional semiconductor devices and will aid performance optimization in future nanoelectronics.

Developing nanoelectronic devices by either top-down engraving approach or bottom-up assembling techniques has garnered much attention, not only because of the interesting optoelectronic properties at the nanoscale but also due to the practical need for mass production in the modern semiconductor industry^1^,^2^,^3^. To manufacture high-performance nanoelectronics with finely controlled charge transport, the electrical properties of the nanocontact (NC) must be seriously considered, particularly in semiconductor nanostructures^4^,^5^,^6^,^7^,^8^. Research on NC resistance traces back more than a decade, when the conductivity of an individual carbon nanotube was mapped using a conducting atomic force microscope^9^. Later, it was found that the contact resistance on carbon nanotubes and semiconductor nanowires (NWs) could be reduced by local heating with an electron beam^10^ or by increasing the thickness of the Ti electrode^11^ To produce Ohmic contacts between NWs and metal electrodes, an ultrasonic nanowelding bonding technique was developed^12^. The electrical properties of NCs in NW devices have been investigated using simultaneous two- and four-terminal conductivity measurements^13^,^14^ and can be analyzed in detail by the transmission-line method^15^. The specific contact resistivity in NCs depends strongly on the NW carrier concentration^16^. It was argued that, as NW diameters shrink, either metal-electrode-induced localization^17^ or Fermi-level pinning^18^ may occur in the interface between the metal electrode and semiconductor NW. Other research has addressed how disorder in the NC alters device electrical properties^19^,^20^. Many possible factors affect the electrical properties of NCs in nanoelectronics, so we must gain a better fundamental understanding of metal/NW contacts.

Indium phosphide (InP) is a III-V semiconductor with a direct band gap of 1.35 eV at room temperature (RT)^21^. This band gap energy is compatible with optical fiber telecommunications and is feasible for optoelectronic applications, and bulk InP is commonly used as a substrate. InP was one of the earliest used semiconductors for achieving NW structures by using a laser-assisted catalytic method^22^. Many proofs of concept were then immediately demonstrated, such as field-effect transistors^23^, polarized photodetectors^24^, waveguides^25^, spin electronics^26^, and solar cells^27^. Both axial^28^ and core-shell heterostructures^29^ based on InP NWs have been made. Recently, calculations have shown that InP NWs with ultrasmall diameters (~2 nm) are highly susceptible to strong surface passivation by H_2_ and O_2_ gases^30^. To clarify how NC electrical properties contribute to device properties, as well as to probe intrinsic carrier behaviors, the electronic transport through individual InP NWs has been studied by analyzing current—voltage (*I*_*ds*_ − *V*_*ds*_) characteristics^31^,^32^,^33^. Meanwhile, using scanning photocurrent spectroscopy, Maharjan *et al.* demonstrated two back-to-back Schottky barriers on InP NW devices^34^. From these experimental results, the underlying mechanism of charge transport in InP NW devices has been described by a number of discrepant models, including thermally activated transport, Mott’s variable range hopping (VRH), and thermionic emission.

Because of their large surface-to-volume ratios and the reduced dimensionality of the active areas, one-dimensional nanostructures, namely NWs, are regarded as promising materials for ultrasensitive sensors. To further enhance the surface effect of NWs, maximizing the change in conductance or resistance, the contacts on the two ends of the NW are chosen with intuition to be Ohmic. In this Letter, we reconsider this kind of arguments. Based on the selected, contact-dominated InP NW devices, we investigated two types of NC resistance, Schottky contact resistance and Mott’s VRH resistance, connected in parallel. A model of Ti/TiO_*x*_ in the interface of the metal electrode and InP NW is developed to explain the parallel connection for both types of NC resistance. Through analyzing the variation in the device resistance over a wide temperature range, we deeply comprehend how both NW and NC resistance contribute to the nanodevice properties. With increases in RT device resistance, both in the NC resistance and the total device resistance, were dominated by Mott’s VRH resistance, supporting the vital role of disorder in the NC. Such disorder NCs were corroborated with cross-sectional transmission electron microscopy (TEM) and the same NC electrical properties also observed in ZnO NW devices, confirming the tenability of this study as well as a universal NC model building. More excitingly, disorder in a NC can be treated as a new functional electronics for carriers, enhancing both the light and gas sensitivity of NW devices.

## Results

**Figure 1:**
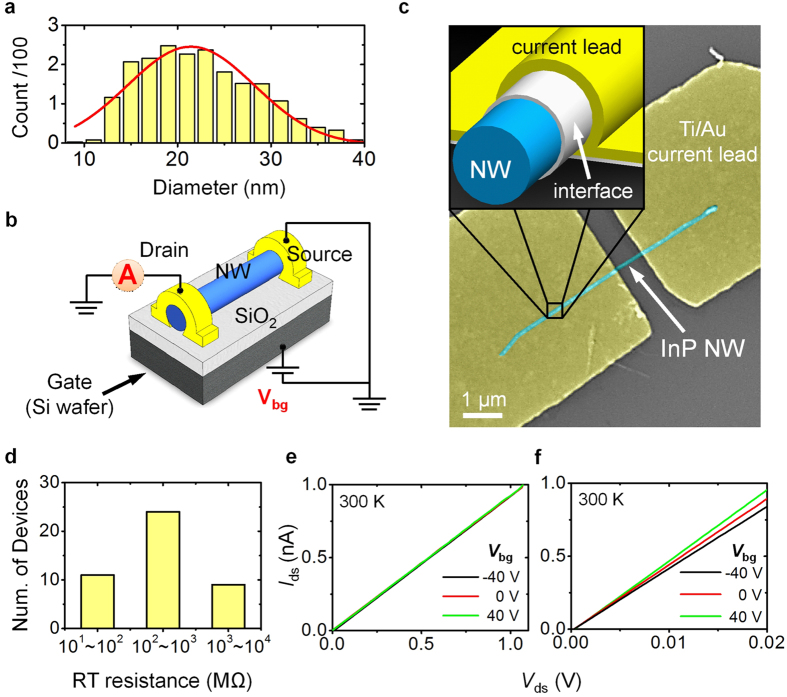
(**a**) Statistical distribution of InP NW diameters fitted with a Gaussian curve in red. (**b**) Schematic of the InP NW device, with overlaid circuit diagram used in this study. A highly doped Si substrate was used as the back gate. (**c**) Microscope image of a typical InP NW device. The inset shows the NC of the Ti layer, defined as the interface between an InP NW and the Au current lead. The thickness of the Ti NC layer was ~20 nm. (**d**) Histogram of RT resistance for the InP NW devices at zero gate voltage. *I*_*ds*_ − *V*_*ds*_ curves under different *V*_*bg*_ for InP NW devices with RT resistances of (**e**) 1070 (NW1) and (**f**) 22 (NW7) MΩ, respectively.

The InP NWs used for this work were synthesized by self-seeded solution-liquid-solid growth and were stored in toluene. The growth details are given elsewhere^35^. To observe the morphology and structure of these NWs, we deposited them onto a carbon-coated grid. Through TEM measurements the NWs that we inspected exhibit straight in the longitudinal direction for several microns, with a few of them showing kink structures. Statistical distribution of the NW diameter is given in Fig. 1a and is fitted by a Gaussian curve, yielding a mean diameter and standard deviation of about 21.4 and 13.5 nm, respectively. For device fabrication, the straight NWs were purposely chosen and the channel length of NW devices was kept a constant of ~1 *μ*m so as to diminish device-device variations. Figure 1b shows a sketch of an InP NW device configuration with the circuit diagram overlaid; the NW is covered by two Ti/Au current leads, regarded as the source and drain electrodes, while a highly doped Si substrate is used as the back gate. (Experimental configurations can be found in Section I of the Supplementary Information.) Fig. 1c displays a microscope image of a typical InP NW device made lithographically. It is noticed that the NC is the interface between the Au electrode and the InP NW, as shown in the inset of Fig. 1c. In the following discussion, we define ***the NC as the Ti layer, which has dimensions of several microns in length,***~***10***–***30 nm in width and***~***20 nm in thickness***.

To study the electrical properties of the NC, we first examined the RT device resistances by using a linear least-squares fit to the *I*_*ds*_ − *V*_*ds*_ curves near zero bias at a back-gate voltage (*V*_*bg*_) of 0 V. Figure 1d summarizes the RT resistances for all the as-fabricated InP NW devices. Unexpectedly, the RT device resistances were broadly distributed, varying from 10^1^ to 10^4^ MΩ. To further investigate the gate-voltage dependence of the *I*_*ds*_ − *V*_*ds*_ output characteristics, we picked two devices with very different RT resistances. Figure 1e and f reveal *I*_*ds*_ − *V*_*ds*_ curves at different *V*_*bg*_ values in the positive *V*_*ds*_ regime for InP NW devices with RT resistances of 1070 (NW1) and 22 (NW7) MΩ, respectively. Sweeping *V*_*bg*_ from − 40 to 40 V, we found the behavior of NW1 (high RT resistance) was independent of gate voltage, whereas the magnitude of *I*_*ds*_ increases at a given *V*_*ds*_ in NW7 (low RT resistance), which suggests that electrons are the majority of charge carriers in InP NWs, indicating *n*-type demeanor. The corresponding transfer characteristics for both NW1 and NW7 devices are shown in Figure S2 in the Supplementary Information. Compared with the data reported previously^23^, the weak gating effect of NW7 may stem from either few carriers in the InP NW or a thick depletion region along the NW diameter. Because we kept the device fabrication constant, such a broad variation in RT resistance and the difference in gating effect support the importance of the NC, which may give rise to a high contribution of contact resistance to NW devices, suppressing carrier injection and intrinsic NW performance.

**Figure 2:**
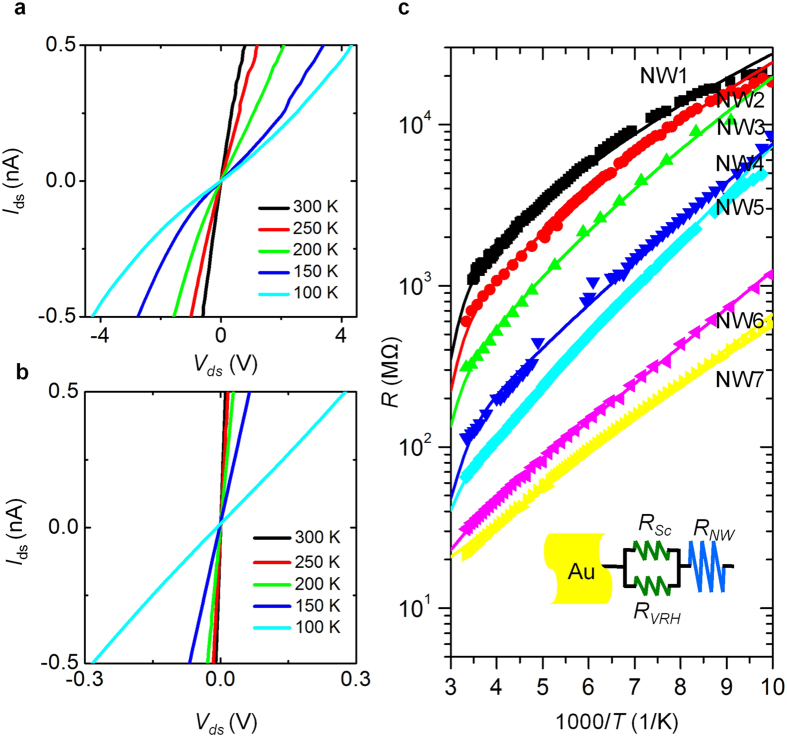
*I*_*ds*_ − *V*_*ds*_ curves at various temperatures for InP NW devices with RT resistances of (**a**) 1070 (NW1) and (**b**) 22 (NW7) MΩ, respectively. (**c**) Resistance as a function of inverse temperature for InP NW devices (NW1–7). The RT resistances are 1070, 650, 312, 99, 65, 31, and 22 MΩ for NW1–7 devices, respectively; the solid lines are best fits. The inset introduces a resistor network model to describe the total device resistance from the NC to the NW. The NC resistance comprises two parallel, connected resistances: the Schottky contact resistance (*R*_*Sc*_) and Mott’s VRH resistance (*R*_*VRH*_). The total device resistance is diagramed as the NC resistance connected with the NW resistance (*R*_*NW*_) in series.

Analysis of *I*_*ds*_ − *V*_*ds*_ characteristics at RT alone cannot give us full understanding of charge transport in nanoelectronics or the microscopic nature of the NC^19^,^20^ Thus, we investigated the temperature dependence of the *I*_*ds*_ − *V*_*ds*_ behaviors for NW1 and NW7, as shown in Fig. 2a,b, respectively. As the temperature decreased from RT to 100 K, a clear nonlinearity appears in the data for NW1 and becomes progressively more pronounced at lower temperatures, while the *I*_*ds*_ − *V*_*ds*_ behavior for NW7 remained linear within a scanned *I*_*ds*_ range of ±0.5 nA. Using these *I*_*ds*_ − *V*_*ds*_ curves, we derived the resistance as a function of temperature for the InP NW devices (NW1–7), as plotted in Fig. 2c. All the resistances increased with decreasing temperature. Unlike the linear variation of temperature dependence on the logarithmic resistance scale for NW7, demonstrating the electrical properties of an intrinsic NW (extensive discussion in Section III of Supplementary Information), NW1 distinctly exhibited a downward bending.

Let us contemplate the mechanism behind this data and build a proper transport model for such temperature dependence (see Fig. 2), because existing models do not reflect the exhaustive behavior of charge transport at the nanoscale, especially in NCs^4^. In our previous report^20^, we discovered both a Schottky contact resistance (*R*_*Sc*_) and Mott’s VRH (*R*_*VRH*_) resistance in the NC. Considering the possibility of total conductivity, an NC can thus be modeled as those two resistances connected in parallel. We argued that the intrinsic carrier transport of the InP NWs can be well described by thermal activation^32^, following the form of *R*_*NW*_(*T*) = *R*_*NW*,0_*exp*(*Ea*/*k*_*B*_*T*), where *k*_*B*_, *E*_*a*_, and *T* are the Boltzmann constant, activation energy, and absolute temperature in Kelvin, respectively. *R*_*NW*,0_ is a constant. To analyze the total device resistance (*R*_*total*_) of the NW device, we merged the NW resistance connected in series with the NC resistance. In the NC, one part of the resistance, the Schottky contact resistance, obeys the mathematical form of *R*_*SC*_(*T*) = (*R*_*Sc*,0_/*T*)*exp*(*q*Φ_*BE*_/*k*_*B*_*T*)^21^, where *q*, Φ_*BE*_, and *RS*_*c*,0_ are the elementary charge, effective Schottky barrier height, and a constant. The other part of the NC resistance, the Mott’s VRH resistance, is expressed by *R*_*VRH*_(*T*) = *R*_*VRH,0*_exp((*T*_0_/*T*)^1/*p*^)^36^, where *p* is an exponent for disordered systems, and *R*_*VRH,0*_ and *T*_0_ are constants. *R*_*total*_(*T*) can be signified as an integrated formula of 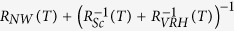. To describe *R*_*total*_(*T*) from the NC to the NW, we introduce an equivalent resistor network model in the inset of Fig. 2c. After performing a nonlinear least-squares fit of this formula to the data for NW1–7 and other NW devices (ZnO NW devices, see Section IV of Supplementary Information), we found that the temperature-dependent resistance conformed well with the best fits over a wide temperature range (solid lines in Fig. 2c); even the corresponding RT resistances dramatically changed, up to two orders of magnitude. The fitting parameters of NW1–7 are listed in Table 1.

From these fitting parameters in Table 1, we observed that the exponent *p* varies from 1 to 4 and the *T*_0_ increases from ~10^2^ to 10^6^ K with an increase of the RT device resistance. The reason of both parameters *p* and *T*_0_ mainly comes from the NC, rather than the NW. The variation could be due to the change of density of states at the Fermi level and the variation of electron’s localization length in the NC. It should be noticed that for variable range hopping condition, the exponent *p* is 2, 3, 4 for a one-, two-, or three-dimensional disordered system. While the *R*_*NW*_ is predominant in the *R*_*total*_, the thermal activation governs the electrical transport, hence the *p* value will be equivalent to unity. That is why the exponent *p* was observed to close to 1 in these devices with low RT resistances, like the cases in NW4–7 as listed in Table 1. To corroborate that the NC disorder indeed exists in such a small volume (about 20 nm × 20 nm × 1 *μ*m) of the NC for carriers passing through it, the localization length and other parameters are examined as follows. We consider the simple case of the three-dimensional Mott’s VRH which gives *p* = 4 in the mathematical expression. The characteristic parameter *T*_0_ is defined by *T*_0_ = 16/*k*_*B*_*g*_0_*ξ*^3^ 36, where *g*_0_ and *ξ* are density of states at Fermi level, and localization length, respectively. Using *T*_0_ = 3.18 × 10^6^ K for the InP NW1 device and *g*_0_ of 6 × 10^18^ eV^− 1^cm^− 3^ 37, the localization length *ξ* is thus derived to be about 2.2 nm, which is not only shorter than all three dimensions of the defined NC and is but also in excellent agreement with the average size of nanocrystalline Ti grains observed in the NC (the detail discussion about the existence of nanocrystlline Ti grain will be given later). Such a short localization length confirms again the formation of disorder system as well as hopping transport in the NC. According to the hopping transport mechanism, the most probable hopping distance 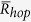 and the average hopping energy 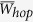 can be calculated from the equations, 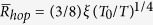 and 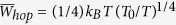. The hopping distance 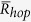 and average hopping energy 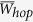 are estimated to be ~10 nm and ~29 meV, respectively, at 100 K. These results strictly satisfy the criteria of 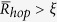 and 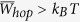 for the Mott’s VRH. Temperature dependent hopping distances and hopping energies are plotted in Figure S5 (see Supplementary Information). The Mott’s VRH explains well the charge transport in the NC at low temperatures and confirms an existence of this special disordered NC system.

**Figure 3:**
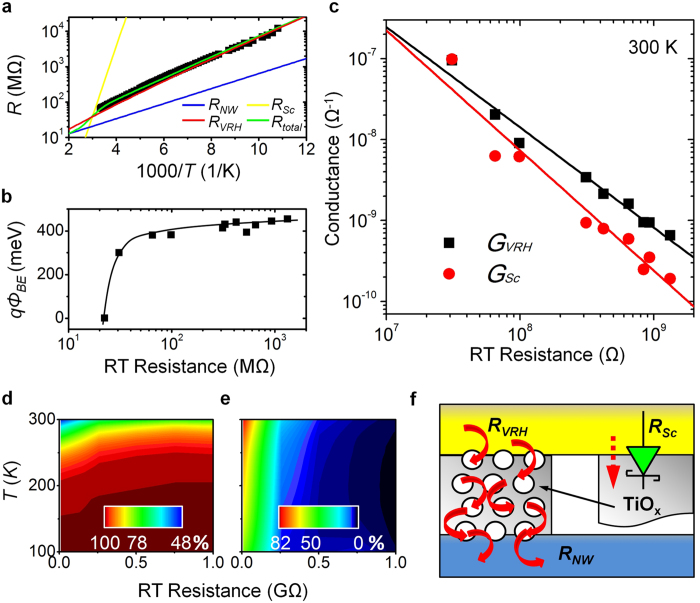
(**a**) Resistance as a function of inverse temperature for NW5 (65 MΩ at RT) with the best fit of *R*_*total*_(*T*) in green. *R*_*NW*_(*T*), *R*_*Sc*_(*T*), and *R*_*VHR*_(*T*) are the fitting results of the intrinsic NW, the Schottky contact and the Mott’s VRH resistances, respectively. (**b**) Effective Schottky barrier height (*q*Φ_*BE*_) as a function of the RT device resistance; the solid line is a guide for the eye. (**c**) The contribution of the two NC conductances, *G*_*Sc*_ and *G*_*VRH*_, at RT as a function of the RT device resistance, where the conductance is a reciprocal value of the resistance. The solid lines are guides for the eye. (**d**) Map of contribution ratio of the Mott’s VRH conductance (*G*_*VRH*_/(*G*_*Sc*_ + *G*_*VRH*_)) as a function of temperature and the device RT resistance. (**e**) Map of contribution ratio of the intrinsic InP NW resistance (*R*_*NW*_/*R*_*total*_) as a function of temperature and the RT device resistance. (**f**) Schematic of the two NC resistances, *R*_*VRH*_ and *R*_*Sc*_. The yellow, gray, white, and blue colors denote Au, TiO_*x*_, Ti, and InP materials, respectively.

To further explore the coincidence between the experimental data and the best fit in detail, we present a typical fit for NW5 in Fig. 3a. The experimental data of the temperature-dependent resistance, shown by filled squares, can be fit well by the *R*_*total*_(*T*) (green line). To identify each contribution to the total device resistance, we import the NW resistance (*R*_*NW*_(*T*), blue line) and the NC resistance, consisting of the Schottky contact resistance (*R*_*Sc*_(*T*), yellow line) and Mott’s VRH resistance (*R*_*VRH*_(*T*), green line) connected in parallel. *R*_*NW*_(*T*) contributes to the *R*_*total*_(*T*) only at higher temperatures; at lower temperatures it drops rapidly and becomes negligible. The magnitude of the *R*_*NW*_(*T*) contribution decreased from ~50% at 300 K to ~10% at 100 K, implying that *R*_*total*_(*T*) in NW5 mainly comes from the NC resistance rather than the NW resistance, especially at lower temperatures. We also examined the contribution of *R*_*Sc*_(*T*) and *R*_*VRH*_(*T*) to the NC resistance. Because the two NC resistances are connected in parallel, the one with lower resistance will contribute more to the total NC resistance. Checking the best fits of *R*_*Sc*_(*T*) and *R*_*VRH*_(*T*), *R*_*VRH*_(*T*) almost dominates the NC resistance, while *R*_*Sc*_(*T*) only contributes near RT. To further scrutinize our observations, we extracted one of the fitting parameters in the *R*_*Sc*_(*T*), the effective Schottky barrier height, as a function of the RT device resistance; this is displayed in Fig. 3b. The NW device with the lowest RT resistance reveals a trivial contribution to the *R*_*Sc*_(*T*), so its effective Schottky barrier height is ignored. The increase in effective Schottky barrier height near several tens of MΩ indicates a formation of an Ohmic contact from the originally pure Ti/Au electrode as well as a Schottky interface in the NC after oxidation caused by poor during metallization (to be discussed later), increasing the RT device resistance. Coinciding with increases in the RT device resistance, the barrier height increased above 300 meV, saturating at ~ 430 meV. This feature demonstrates that *R*_*Sc*_(*T*) immediately affects the NC electrical properties for a device with low RT resistance, but quickly becomes invariable as the RT device resistance increases up to 1 GΩ. These results suggest that *R*_*Sc*_(*T*) alone could not have caused the major increases in device resistances, corroborating the best fits shown in Fig. 3a and the integrated NC model capable of describing the temperature-dependent behaviors.

Because the two NC resistances, *R*_*Sc*_(*T*) and *R*_*VRH*_(*T*), are connected in parallel, we use their reciprocals (conductances) to more clearly show their contributions. Figure 3c manifests the Schottky contact conductance (*G*_*SC*_ = 1/*R*_*SC*_) and Mott’s VRH conductance (*G*_*VRH*_ = 1/*R*_*VRH*_) at 300 K of the NC as functions of the RT device resistance. For devices with a low RT resistance, *G*_*Sc*_ is comparable with *G*_*VRH*_. As the RT device resistance increased, *G*_*VRH*_ gradually surpassed *G*_*Sc*_ and dominated the total device resistance. At 300 K, *G*_*VRH*_ contributed more than 70% for devices with a high RT resistance of ~0.5 GΩ. Figure 3d maps the contribution ratio of *G*_*VRH*_/(*G*_*Sc*_ + *G*_*VRH*_) as a function of temperature and the RT device resistance. The ratio of *G*_*VRH*_ contribution reveals that the disorder effect entirely governed the whole NC, up to 100% at temperatures lower than 150 K. Even at 300 K, it contributed 50%–75%, increasing with the RT device resistance. This result again supports that the increase in NC resistance and total device resistance primarily came from Mott’s VRH, pointing toward the dominance of the disorder effect in the NC. However, the intrinsic NW resistance depends on the NC resistance in series. Thus, Fig. 3e maps its contribution ratio as a function of temperature and the RT device resistance: *R*_*NW*_/*R*_*total*_. At 300 K, the contribution of *R*_*NW*_ decreased rapidly, from almost 100% for devices with a RT resistance of 22 MΩ to insignificant for devices with a RT resistance larger than 0.5 GΩ. More specifically, at 100 K, *R*_*NW*_ contributed less than 40% to *R*_*total*_. *By systematically analyzing the temperature dependence of the electrical characteristics, we conclude that R*_*NW*_ *contributes mainly at RT for devices with low RT resistance, while at low temperatures the NC dominated the total device resistance. At low temperatures, R*_*VHR*_ *and the disorder effect dominated both the NC resistance and the total device resistance.*

We will now speculate on the reason of the integrated transport model for the total device resistance. In our setup, the two ends of the InP NWs were covered by Ti/Au (source and drain) electrodes, and the thickness of the deposited Ti layer was about 20 nm. Because the electron affinity of bulk InP is ~ 4.4 eV^38^, close to the work function of 4.33 eV for Ti metal^21^, a perfect InP/Ti interface will have Ohmic behavior rather than Schottky behavior. In contrast, it has been argued that titanium oxide could form easily during thermal evaporation of a Ti layer (extensive demonstration in Section VI of Supplementary Information)^20^,^39^. The electron affinity of TiO_*x*_ is 4.33 eV^40^, and the work function of Au is 5.1 eV^21^. Therefore, we infer that a Schottky contact developed on the interface of TiO_*x*_/Au. However, the disorder system of Mott’s VRH resistance have been caused by granular Ti metal clusters embedded in the related insulting matrix^20^. Figure 3f depicts such an idea of the NC resistance; the right and left sections explain a possible Schottky contact resistance and Mott’s VRH resistance in the NC, respectively. It is worth noting that the ideal Schottky barrier, the energy difference between the electron affinity of TiO_*x*_ and the work function of Au, is 770 meV, larger than the saturated effective barrier height of 430 meV estimated experimentally (see the discussion of Fig. 3b). This discrepancy in barrier height may have originated from imaging force lowering, the density distribution of interface states, or traps within the space-charge region; further discussion is needed. In short, a disorder layer appears to exist at the interface and can be modulated to show Ohmic behavior, Schottky behavior, or disordered NCs on the InP NWs. To avoid the formation of TiO_*x*_ in the NC during metallization is critical for investigating intrinsic charge transport of NWs. The dramatic variation in electrical properties of the NW devices can be determined by our integrated transport model, created from temperature-dependent studies.

The high RT device resistance was caused by a poor NC during Ti electrode deposition, and the disorder introduced in the NC causes additional energy dissipation, leading to bad device performance, such as decreased ambient sensitivity in sensors. Regardless, we demonstrated that the electrical properties of the NW devices dominated by the disorder effect (Mott’s VRH) can be beneficial for developing new electronic functions in nanoelectronics; for example, by modifying light and gas sensitivities. In the following discussion, we roughly group the InP NW devices into two types, NW- and NC-dominated devices, according to their total device resistance at RT.

**Figure 4:**
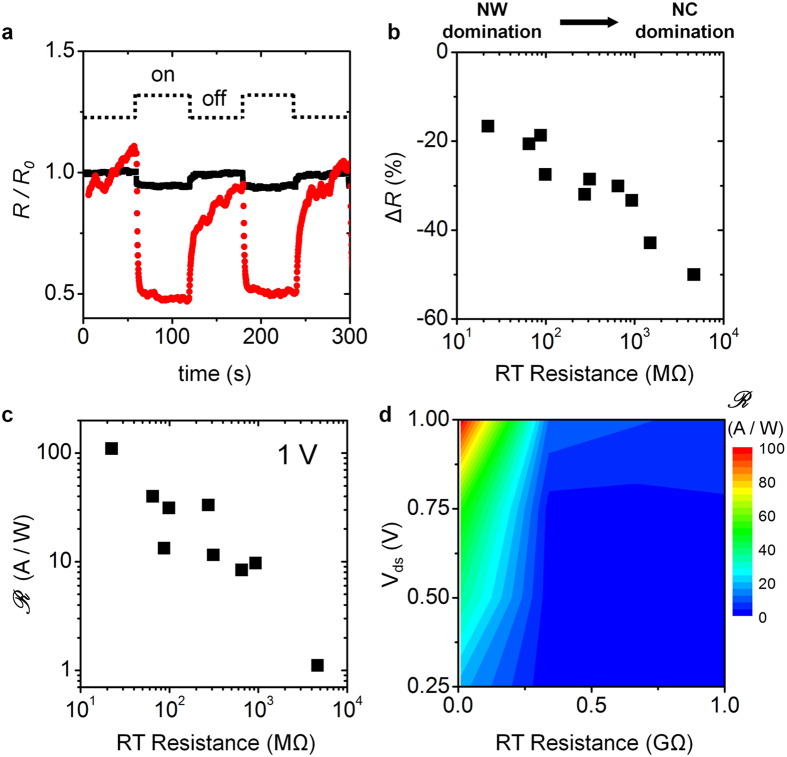
(**a**) Change in resistance in green light switching on and off for NW- (black squares) and NC-dominated (red circles) devices with RT resistances of 22 and 4600 MΩ, respectively. (**b**) Sensitivity as a function of the RT device resistance. (**c**) Responsivity as a function of the RT device resistance. (**d**) Map of responsitivity as a function of *V*_*ds*_ and the RT device resistance.

Figure 4a shows the change in resistance (*R*/*R*_0_) of both NW-dominated (black squares) and NC-dominated (red circles) devices in response to switching a green laser on and off. Here, the resistances before and after applying control variables are labeled *R*_0_ and *R*, respectively. Because the increase in current during light exposure is associated with a decrease in resistance, a decreasing change lower than a unit is expected. Though a time lag occurred in the resistance variation of the NC-dominated device when switching off the light, the change in resistance adequately followed the switching. We define the sensitivity as ∆*R* = (*R* − *R*_0_)/*R*_0_; it is extracted in Fig. 4b as a function of the RT device resistance. Surprisingly, increasing the RT device resistance increased sensitivity. As discussed previously, Mott’s VRH resistance and the NC disorder contributed most to the total resistance for the NC-dominated devices. We attribute this trend of enhanced sensitivity to introduction of the disorder effect into the NC. However, as mentioned before, the disordered NC will cause additional energy to dissipate when carriers pass through; the enhanced sensitivity does not violate that energy dissipation principle. The NC disorder improves light sensitivity, but still reduces the photo-induced current without contradicting its characteristic of carrier scattering; evidence for this is shown in Fig. 4c. We define the responsivity as the ratio of photo-induced current to incident light power. The responsivity decreased from ~10 A/W for the NW-dominated device to ~1 A/W for the NC-dominated device. These results show a trade-off between the sensitivity and responsivity due to the NC disorder effect (further discussion in Section VII of Supplementary Information). Figure 4d shows a map of the responsivity as a function of *V*_*ds*_ and the RT device resistance. The responsivity decreased with greater disorder in the NC (or greater RT device resistance), while it increased with greater applied *V*_*ds*_. In other words, the responsivity decreased much faster with stronger disorder in the NC at high *V*_*ds*_.

**Figure 5:**
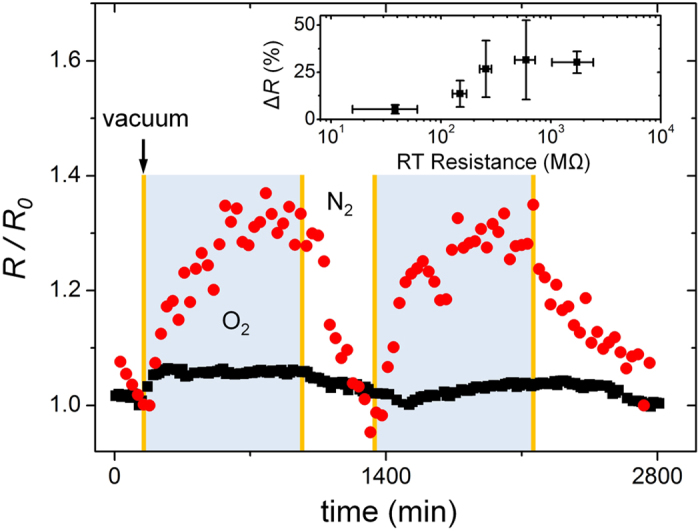
Change in resistance with exposure to oxygen and nitrogen gases for NW- (black squares) and NC-dominated (red circles) devices with RT resistances of 29 and 692 MΩ, respectively. Regions of oxygen exposure for 800 min and nitrogen exposure for 300 min are colored in blue and white, respectively, and the region of vacuum exposure for 20 min is colored in orange. The inset shows the oxygen sensitivity as a function of the RT device resistance.

In addition to exploring light sensitivity, we also investigated the gas sensitivity of these InP NW devices. As shown in Fig. 5, we examined both the NW-dominated (black squares) and NC-dominated (red circles) devices for their response to gas exposure. Compared with the light sensor (Fig. 4a), much more time is required to inspect the gas response because of the diffusion mechanism of the injected gas and oxidation at the surface of InP NWs. For the NW-dominated device, its resistance ratio changed in O_2_ with regard to that in N_2_ is ~10%, which closely matches that of 8% in its thin film^41^, confirming again that its electrical behavior was dominated by the intrinsic InP-NW channel rather than by the disordered NC. In contrast, the change in resistance for the NC-dominated device increased much more than that of the NW-dominated one. This is the first experimental verification that InP NWs can be used in gas nanosensors. The inset of Fig. 5 summarizes the sensitivity induced by O_2_ as a function of the RT device resistance. Similar to the light sensitivity of the InP NW devices, as the RT device resistance or NC disorder increased, the O_2_ sensitivity gradually increased. The O_2_ sensitivity seemed to saturate at ~ 30% for devices with RT device resistance higher than ~ 0.5 GΩ.

**Figure 6:**
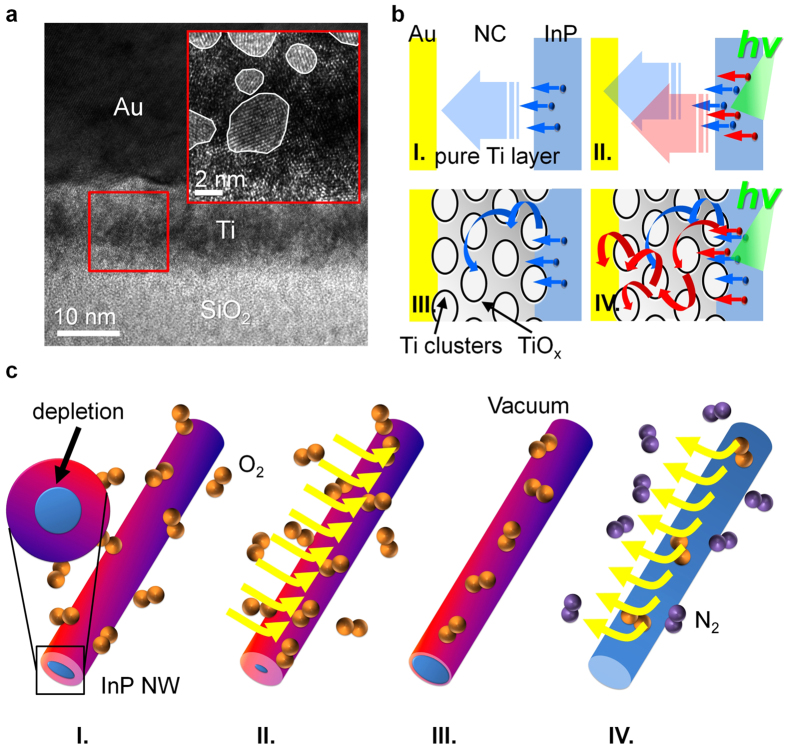
(**a**) Cross-sectional TEM image of the SiO_2_/Ti/Au interface. The specimen was prepared by the same thermal-evaporation method used to fabricate the InP NW devices. The inset shows a magnified view of the square area, revealing crystalline Ti metal clusters embedded in a disordered layer. (**b**) Schematic of charge transport for the NC of the NW-dominated device (I) in the dark and (II) in green light, and schematic of charge transport for the NC of the NC-dominated device (III) in the dark and (IV) in green light. (**c**) Schematic of gas exposure in (I and II) oxygen, (III) vacuum, and (IV) nitrogen.

To consolidate the existence of the NC disorder, we used cross-sectional TEM for imaging and spectroscopy in the region of interest, particularly the NC structure. Because thermal evaporation is a benign process, not damaging the NW crystallinity at the surface, Fig. 6a shows a cross-sectional TEM image of the SiO_2_/Ti/Au interface (without InP NWs) prepared with the same metallization as in previous samples. This figure reveals insulating SiO_2_, Ti, and Au layers stacked in sequence, as well as bright, spotty contrast distributed in the whole Ti layer, which seems to reflect an ordered, crystalline lattice structure in the Au layer. Using high-resolution TEM, shown in the inset of Fig. 6a, we found that this spotty contrast in the Ti layer is nanocrystallites (average size of ~ 2 nm) embedded in a disorder matrix. Analyzing these nanocrystallites with a fast Fourier transform, we found a dominant *d*-spacing of ~ 0.22 nm in the diffraction ring (not shown here), consistent with pure crystalline Ti, corroborating the presence of NC disorder. Besides, the reasonable size of Ti clusters is in high accordance with the estimated localization length of the hopping transport, giving additional evidence for Mott’s VRH.

Finally, to interpret the difference in sensitivity between the NW- and NC-dominated devices, we propose a transport mechanism for both light and gas sensors. For the light sensors, when the incident light impinges on the NW, the generated excited currents produce a signature higher energy than the dark currents. These photo-induced currents are driven by applied *V*_*ds*_ to overpass the NC. We must restate that we define the NC as the Ti layer between the Au electrode and the InP NW. In the NW-dominated devices, the change in resistance is determined from the intrinsic photo-induced currents of the InP NW (see Fig. 6b, parts I and II). But, when accounting for the effect of NC disorder, the resistance change will differ completely from the intrinsic value. We modeled and experimentally confirmed the NC disorder as granular Ti metal clusters embedded in a disorder matrix. Because the hopping possibility is proportional to 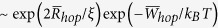
36, the presence of smaller size of these metallic Ti clusters involves a diminished current flow of low-energy carriers passing through the NC. In this case, the dark currents will be filtered out, whereas the photo-induced currents will be bypassed through the disordered NC. The NC disorder increases the difference in hopping probability between dark and photo-induced currents, so the light sensitivity increases as the RT device resistance increases. This concept of the disorder filter is shown in parts III and IV of Fig. 6b. Similarity, Fig. 6c shows the operational processes of the InP NW gas sensors. When O_2_ was introduced to the surface of the InP NW, the oxidation process traps carriers to form a depletion region, increasing the RT device resistance (Fig. 6c, part I). As the O_2_ gas diffuses further into the NW, the depletion region enlarges, increasing the gas sensitivity to its maximum, as shown in part II of Fig. 6c. When the device is put in vacuum, this removes O_2_ molecules from the NW surface (Fig. 6c, part III). As the device is put in N_2_ gas, O_2_ molecules diffuse out extensively from the InP NW (Fig. 6c, part IV). Because the oxidation changes the surface Fermi level of NWs^42^, it is rational that the carriers at the surface and those deep in the NWs have different energies. Thus, for a NC-dominated device, the disorder in nanoelectronics can enhance the gas sensitivity.

## Discussion

In conclusion, we provided in-depth understanding of the charge transport behavior for ***common semiconductor NW devices***, especially on the dominant NC mechanism at the mesoscopic scale. To accurately describe the temperature-dependent resistance of NW devices, we developed a generalized, integrated transport model that considers the intrinsic NW resistance following thermal activation as well as the NC resistances connecting the Schottky contact resistance and the Mott’s VRH resistance in parallel. As the RT device resistance increased, the effective Schottky barrier height increased rapidly above 300 meV and then saturated to an average of 430 meV, whereas the contribution of the Mott’s VRH resistance gradually grew and finally dominated both the NC resistance and the total device resistance, indicating the crucial role of NC disorder in charge transport. To explain the parallel connection of the Schottky contact resistance and the Mott’s VRH resistance, we proposed a model of Ti/TiO_*x*_ on the interface between the metal electrode and the NW. NC disorder is beneficial, as it filters and bypasses low- and high-energy carriers, respectively; moreover, NC-dominated devices exhibited enhanced light and gas sensitivities. Understanding the conduction mechanism in NCs is most important for building reliable nanoelectronics, and our temperature-dependent analysis gives insight into NC electrical properties, offering a new way to design electronic functions at the nanoscale. This discovery greatly impacts the fields of nanoelectronics and nanotechnology.

## Methods

### NW Device Fabrications

A heavily p-type doped Si (100) wafer with a resistivity of 0.002 Ω-cm was used as a substrate. For measurements of electric-field effect characteristics, this wafer substrate was taken as a back gate electrode. Before the dispersion of InP NWs on the Si substrate, a SiO_2_ layer with an average thickness of ~ 400 nm was capped on the substrate by thermal oxidation to prevent the on-chip devices from any current leakages through the substrate. Afterward, micrometer-scale connection lines, markers, and sub-millimeter electrode pads for electrical probing were patterned on the substrate by photo-lithography and thermal evaporation of Ti/Au thin films with ~ 10/60 nm in thickness. The suspensions of InP NWs in toluene were then drop-cast to disperse the NWs on the patterned substrate. Prior to metallization procedure, the substrate with bare InP NWs was heated in a high vacuum at 150 °C for 12 h to remove any contaminations such as capping ligands on surface of these NWs. The position of these NWs was determined by microscope images. Subsequently, a standard electron-beam lithography and thermal evaporation were adopted to make two Ti/Au current leads (~ 20/100 nm in thickness) on an individual NW, connecting to the large pattern (micrometer-scale electrodes). Here, the separation distance between two (source and drain) electrodes on the InP NW was purposely maintained as a constant of ~1 *μ*m. To improve the contact, these NW devices fabricated were heated again in a high vacuum up to 400 °C with a rate of 10 °C/min, annealed for 1 min, and cooled down to RT. The layout of patterned electrode on the Si wafer as well as the detailed configuration of NW devices is schematically illustrated in Figure S1.

### Electrical properties

Current-voltage (*I*_*ds*_ − *V*_*ds*_) curves, having current and voltage resolutions of 10 pA and 1 mV, respectively, were measured using an electrometer (Keithley K6430) and programmed by the LabVIEW software. The values of device resistances were calculated through a linear least square fit to the *I*_*ds*_ − *V*_*ds*_ curves near the zero bias at the back-gate voltage of 0 V and their standard deviation was evaluated to be less than 0.1%. For acquiring temperature dependence of electrical properties, the NW devices were loaded into a cryostat (Variable Temperature Inert Cryostat, CRYO Industries of America Inc.) in 1-atm helium (99.99%). The temperature was precisely monitored within the range from 300 to 80 K using a LakeShore 340 temperature controller. On the other hand, the NW devices were also placed in a vacuum chamber for characterizing light- and gas- responses. For light sensing, photo-induced current was excited using a green light with a wavelength of 532 ± 0.15 nm, a power of 20 mW, and a beam size of 2 mm in diameter. The light intensity was estimated to be about 0.5 W/cm^2^. For gas sensing, the chamber was continually put in 1 atm oxygen, a vacuum, 1 atm nitrogen, and a vacuum again to observe resistance variations of a InP NW device.

## Additional Information

**How to cite this article**: Lin, Y.-F. *et al.* Nanocontact Disorder in Nanoelectronics for Modulation of Light and Gas Sensitivities. *Sci. Rep.* **5**, 13035; doi: 10.1038/srep13035 (2015).

## Supplementary Material

Supplementary Information

## Figures and Tables

**Table 1:** RT resistances and fitting parameters of InP NW devices (NW1–7).

	RT resistance (MΩ)	RT contact resistance= *R*_*total*_ − *R*_*NW*_ (MΩ)	*p*	*T*_0_ (K)	*q*Φ_*BE*_ (meV)	*E*_*a*_ (meV)
NW1	1070	1048	4	3.18 × 10^6^	444	42
NW2	650	628	3	1.64 × 10^5^	467	42
NW3	312	290	2	9.61 × 10^3^	412	42
NW4	99	77	1.33	1.41 × 10^3^	381	42
NW5	65	43	1.13	1.07 × 10^3^	397	42
NW6	31	9	1.04	6.64 × 10^2^	299	42
NW7	22	N/A	1	N/A	N/A	42
