# Supplementary material for: Nanocontact Disorder in Nanoelectronics for Modulation of Light and Gas Sensitivities
Source: Sci Rep. 2015 Aug 11;5:13035. doi: 10.1038/srep13035 (PMC4531317; doi:10.1038/srep13035)
Supplement: Supplementary Information [file srep13035-s1.doc]

Electronic Supplementary Information

**Nanocontact Disorder in Nanoelectronics for Modulation of Light and Gas Sensitivities**

| Yen-Fu Lin,^∗,†^ Chia-Hung Chang,^‡^ Tsu-Chang Hung,^‡^ Wen-Bin Jian,^∗,‡^ Kazuhito Tsukagoshi,^¶^ Yue-Han Wu,^§^ Li Chang,^§^ Zhaoping Liu,^∥,⊥^ and Jiye Fang^∥^  *∗*To whom correspondence should be addressed.  †Department of Physics, National Chung Hsing University, Taichung, 40227, Taiwan  ‡Department of Electrophysics, National Chiao Tung University, Hsinchu 30010, Taiwan  ¶WPI Center for Materials Nanoarchitechtonics (WPI-MANA), National Institute for Materials Science (NIMS), Tsukuba, Ibaraki 305-0044, Japan  §Department of Materials Science, National Chiao Tung University, Hsinchu 30010, Taiwan  *∥*Department of Chemistry, State University of New York at Binghamton, Binghamton, New York 13902-6000, USA  *⊥*Current address: Ningbo Institute of Material Technology and Engineering, Chinese Academy of Science, Ningbo, Zhejiang, 315201, People’s Republic of China  E-mail: [yenfulin@nchu.edu.tw](mailto:yenfulin@nchu.edu.tw); [wbjian@mail.nctu.edu.tw](mailto:wbjian@mail.nctu.edu.tw) |
| --- |

1. **The experimental configureations.**
2. **Transfer characteristics for InP NW1 and NW.**
3. **The intrinsic electrical properties of InP NWs.**
4. **The universal nanocontact model and validity of the fitting formula.**
5. **Hopping parameters as a function of temperatures.**
6. **Electrical properties of Ti thin films deposited under different pressures.**
7. **Responsivity and quantum efficiency of NW devices.**
8. **The experimental configurations.**


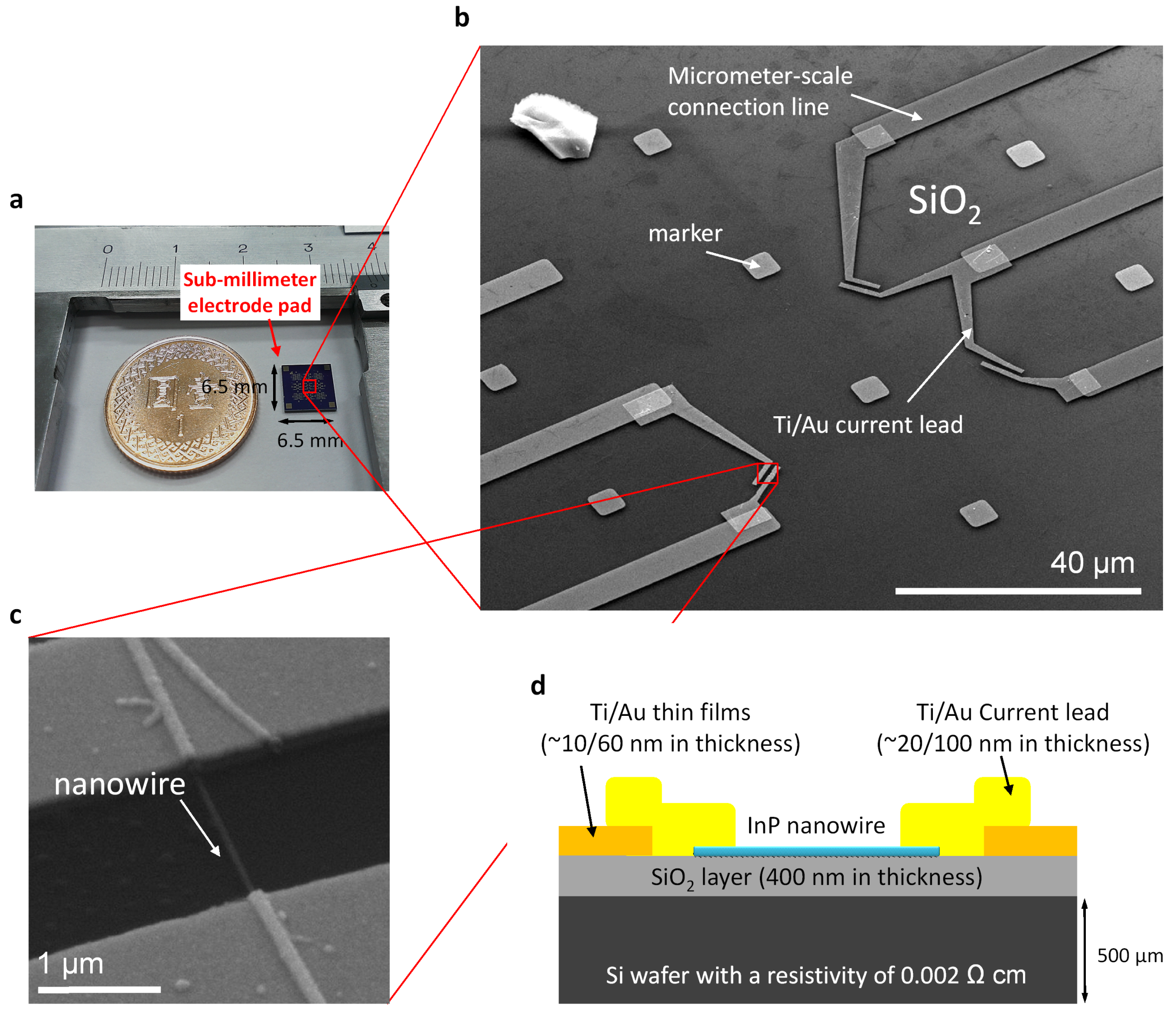


**Figure S1:** (**a**) Photograph of devices on a substrate with a dimension of 6.5 × 6.5 mm^2^ (on the right part of the photography). The left part of the graph displays a copper coin of one New-Taiwan dollar for comparison. The marked rectangle is magnified by microscope and shown in **b**. (**b**) Low-magnification SEM image of three InP NW devices with micrometer-scale connection lines, alignment marker, and current leads. (**c**) High-magnification FESEM image displaying one device of a single InP nanowire embedded under two Ti/Au current leads, where the separation distance between edges of the two current leads is kept at a constant of ~1 μm. (**d**) Scheme of side view of the InP NW FET device. Here the Si wafer with a resistivity of ~0.002 Ω−cm was used as a substrate as well as a back gate electrode for gating effect measurements.

1. **Transfer characteristics for NW1 and NW7.**


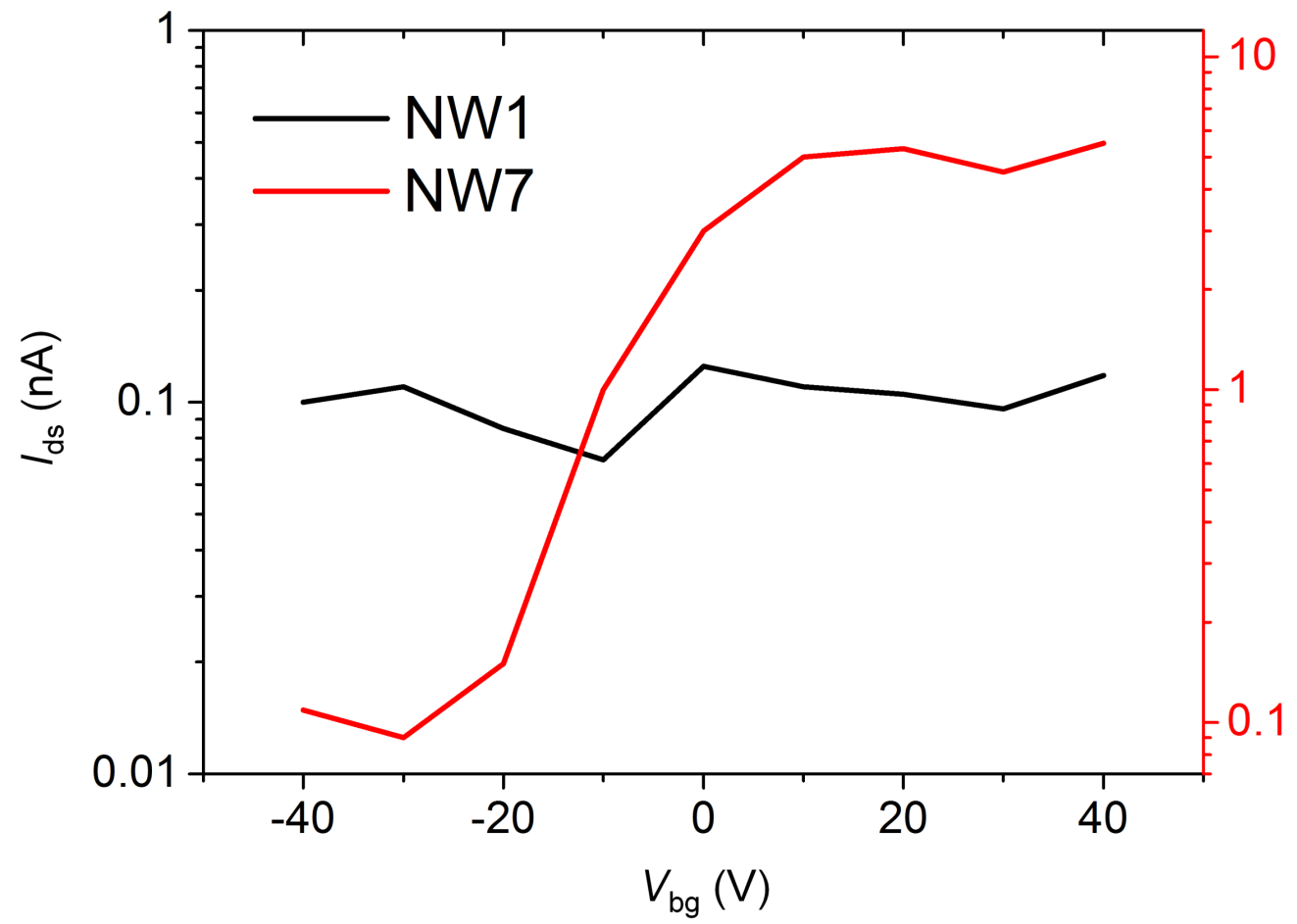


**Figure S2:** Transfer characteristics for InP NW devices with RT Resistance of 1070 (NW1) and 22 (NW7) MΩ, respectively.

1. **The intrinsic electrical properties of InP NWs.**

In our previous report, both four- and two-probe electrical measurements are used to explore electron transport of semiconductor nanowires in a wide temperature range from 300 to 10 K.**^[S1]^** It was found that the intrinsic electron transport of InP NWs can be well-described by the theory of thermal activated transport according to the form $R_{NW}(T)=R_{NW,0}\exp(E_{a}/k_{B}T)$ at high temperatures (100-300 K). However, the electron transport changes to agree with the 3D-Mott’s VRH theory of the form $R_{3D-VRH}(T)=R_{3D-VRH,0}\exp((T_{0}/T)^{1/4})$ at low temperatures. Both of the two transport mechanisms shall be considered simultaneously for explanation of the temperature dependent resistance of intrinsic InP NWs. We thus propose that the intrinsic resistance of InP NWs, $R_{total-NW}(T)$, is composed of two resistances, the thermal activated (*R_NW_*) and the 3D-Mott’s VRH (*R_3D-VRH_*) resistances connected in parallel. The total nanowire resistance, $R_{total-NW}(T)$, is described by the mathematical formula of $(R_{NW}^{-1}(T)+R_{3D-VRH}^{-1}(T))^{-1}$. For example, ***NW7*** shows intrinsic electrical properties of the InP NWs, as described in the main text. In this supplementary information, the device resistance in a wide temperature range is displayed in **Figure S3** with the best fit (in red) of $R_{total-NW}(T)$. It is emphasized again that the InP NW of this device was ~1 μm in length, ~20 nm in diameter, and ~0.69 Ω-cm in resistivity. To identify the contribution in different temperature regime, the fitting results of *R_NW_*(*T*) and *R_3D-VRH_*(*T*) resistances are drawn in blue and green, respectively. The thermal activated transport contributes mainly at a temperature above 50 K while the 3D-Mott’s VRH dominates the device total resistance at lower temperatures. The parameter *T_0_* in formula *R_3D-VRH_*(*T*) was estimated to be about 3.75 × 10^2^ K. From the fitting result, the activation energy, *E_a_*, was estimated to be about 42 meV. Since InP NWs have the same diameter and are intentionally picked up from the same sample base for device fabrications, they shall possess the same order of magnitude of defect concentration as well as NW resistances. Consequently, the *E_a_* parameter was kept as a constant when fitting by the complete, integrated formula of $R_{NW}(T)+(R_{Sc}^{-1}(T)+R_{VRH}^{-1}(T))^{-1}$in the main text.


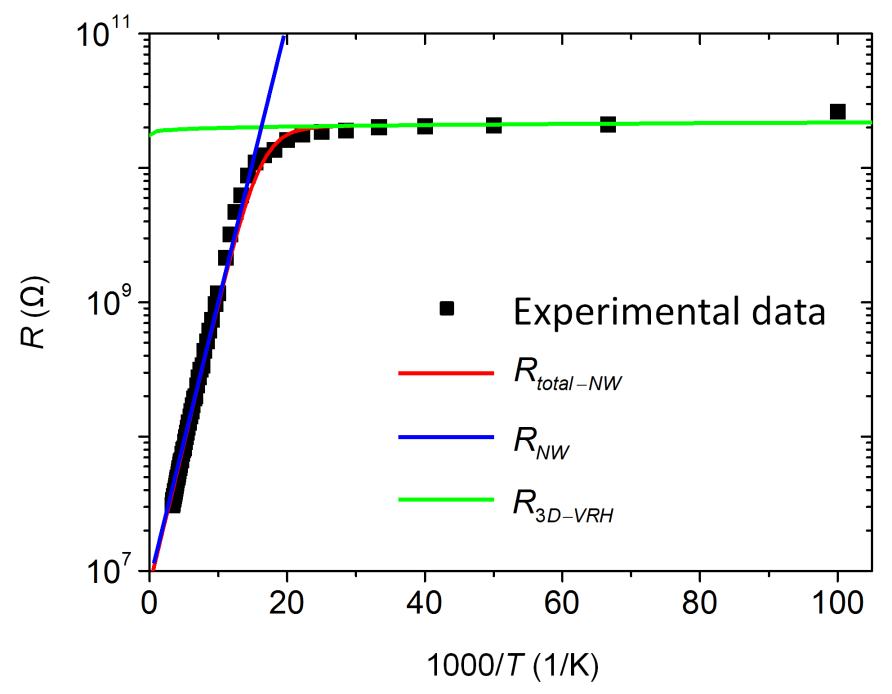


**Figure S3:** Resistance as a function of inverse temperature for NW7 (22 MΩ at RT) with the best fit of $R_{total-NW}(T)$ in red. *R_NW_*(*T*) and *R_3D-VRH_*(*T*) are fitting results of the thermal activated and the 3D-Mott’s VRH resistances, respectively. The total nanowire resistance, $R_{total-NW}(T)$, consists of two resistances, *R_NW_*(*T*) and *R_3D-VRH_*(*T*), connected in parallel.

1. **The universal nanocontact model and validity of the fitting formula.**

To check the universal properties of the NC and the validity of the fit formula of $R_{NW}(T)+(R_{Sc}^{-1}(T)+R_{VRH}^{-1}(T))^{-1}$, we performed the same fabrication and data analysis on ZnO NWs. We fabricated ZnO NW devices by the same fabrication procedure, referring to **Section I in Supplementary Information**. The descriptions of material syntheses, physical properties, characterizations, and fabrications in detail are given elsewhere.**^[S2]^** Temperature dependent resistances of three typical ZnO NW devices are displayed in **Figure S4**. The symbols and solid curves are experimental data and the best fits by using the integrated formula of $R_{NW}(T)+(R_{Sc}^{-1}(T)+R_{VRH}^{-1}(T))^{-1}$. ZnO-3, revealing intrinsic properties of the NW, possesses the lowest RT resistance of 0.02 MΩ. Its transport behavior highly coincides with the theoretical model of the semiconductor transport – the thermal activation form $R_{NW}(T)$, and the activation energy *E_a_* of 110 meV is very close to that obtained from the bulk ZnO. The fitting parameters estimated from the three ZnO NW devices are summarized in **Table S1** The *T*_0_ values are in the same range as those obtained from InP NW devices. The saturated magnitude of the effective Schottky barrier height $q\Phi_{BE}$, which describes another NC property, is also very close to that listed in **Table 1** in the main text. These results confirm that the NC on either InP or ZnO NWs exhibits the same physical properties. In addition, *the flawless fit in* ***Figure S4*** *supports again that the integrated formula can be widely applied to the explanations of electrical properties of NC in nanoelectronic devices.*


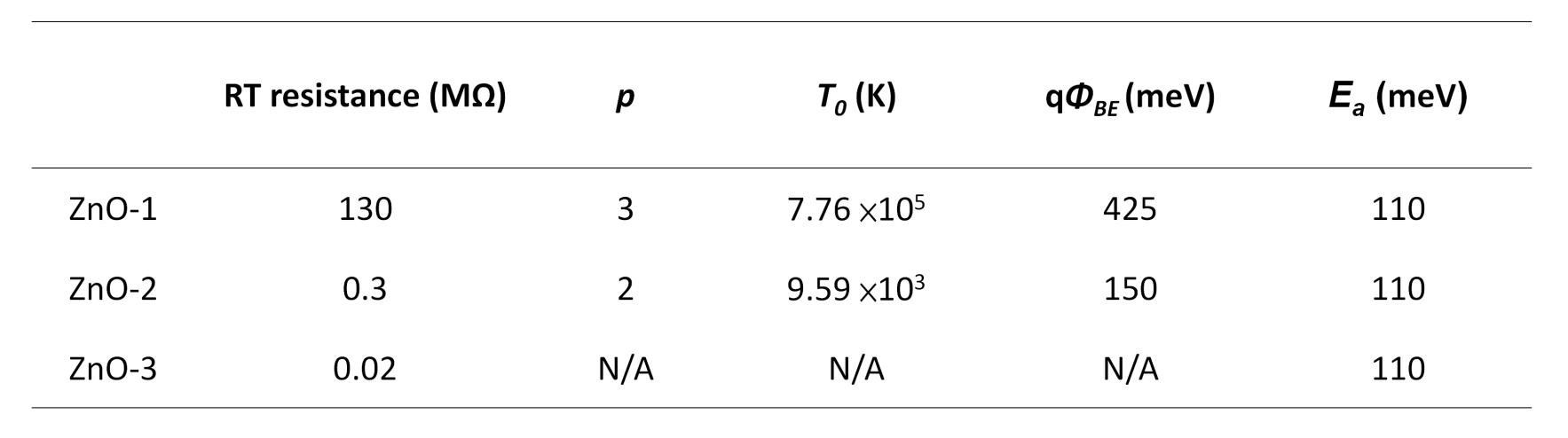


**Table S1:** RT resistances and fitting parameters of ZnO NW (ZnO-1 – ZnO-3) devices.


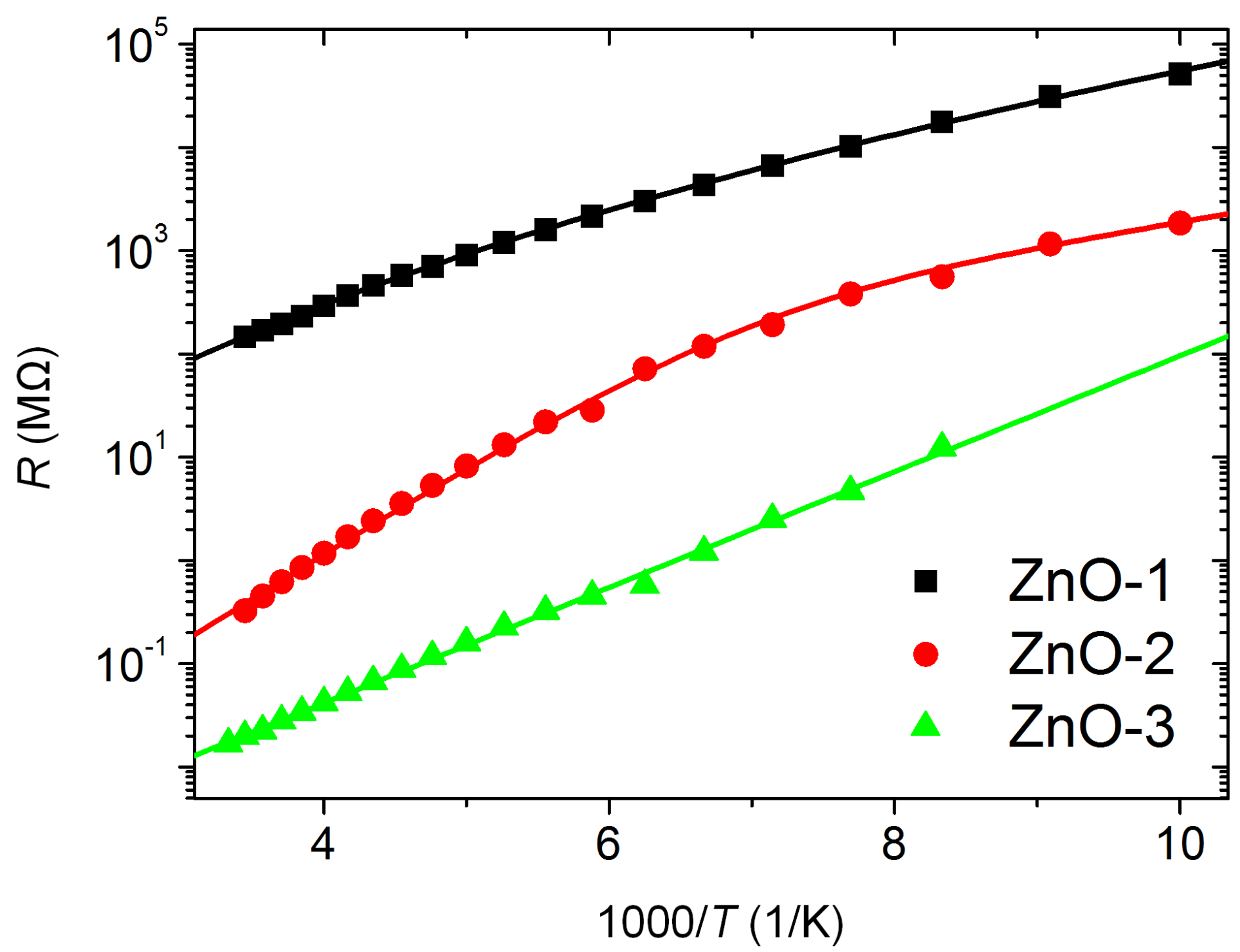


**Figure S4:** Resistances as a function of inverse temperature of three ZnO NW devices. The RT resistances are 150, 0.3, and 0.02 MΩ for ZnO-1, ZnO-2, and ZnO-3. The symbols and solid curves represent experimental data and the best fits, respectively.

1. **Hopping parameters as a function of temperature.**

**
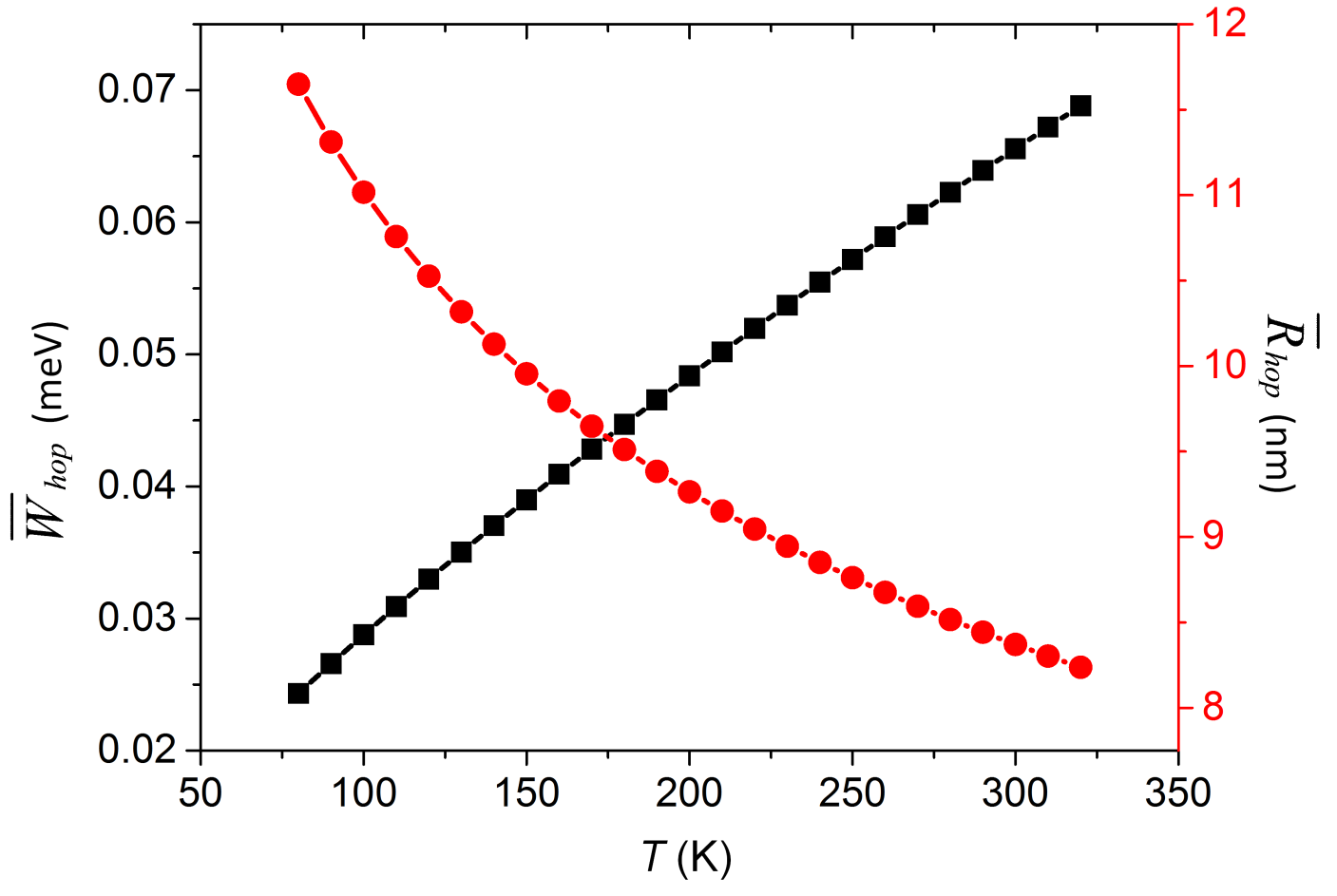
**

**Figure S5:** Hopping energy and hopping distance as a function of temperature for the case with *T_0_*=3.18$\times$10^6^ K.

1. **Electrical properties of Ti thin films deposited under different pressures.**

To verify that titanium oxide could form in the nanocontact due to poor vacuum conditions during thermal evaporation, rendering non-metallic behaviors, the nanogap of ~50 nm in width and ~50 nm in length was patterned by electron beam lithography on a silicon wafer capped with ~400 nm thick SiO­_2_ layer. The experimental details are given elsewhere.**^[S3]^** Ti (~50 nm in thickness) thin films were then thermally deposited under a different base pressure on top of such patterned wafers. To reduce further oxidation on the surface of as-deposited Ti thin films, the samples were spin-coated with ~400 nm thick PMMA 950 A8 layer. Subsequently, these thin-film devices were loaded in a high vacuum (~10^-6^ Torr) for electrical measurements. Resistivity as a function of inverse temperature for two Ti thin films deposited under different base pressures of 10^-5^ (black square) and 10^-6^ (red circle) Torr are presented in **Figure S6**. With a decrease of temperature, the resistivity increases by 2~3 orders of magnitude for the thin film deposited under a poor vacuum condition, demonstrating a non-metallic or semiconducting temperature behavior, whereas that of the thin film deposited under a base pressure of ~10^-6^ Torr decreases monotonically. The RT resistivity of metallic Ti thin film is close to the properties of single-crystalline Ti reported previously.**^[S4]^** Such a dramatic variation in resistivity manifests that titanium oxide could form in a poor vacuum condition which results in the possibility of granular Ti metal clusters embedded in the related insulating TiO_x_ matrix, further dominating electrical transport properties in nanoelectronics.

**
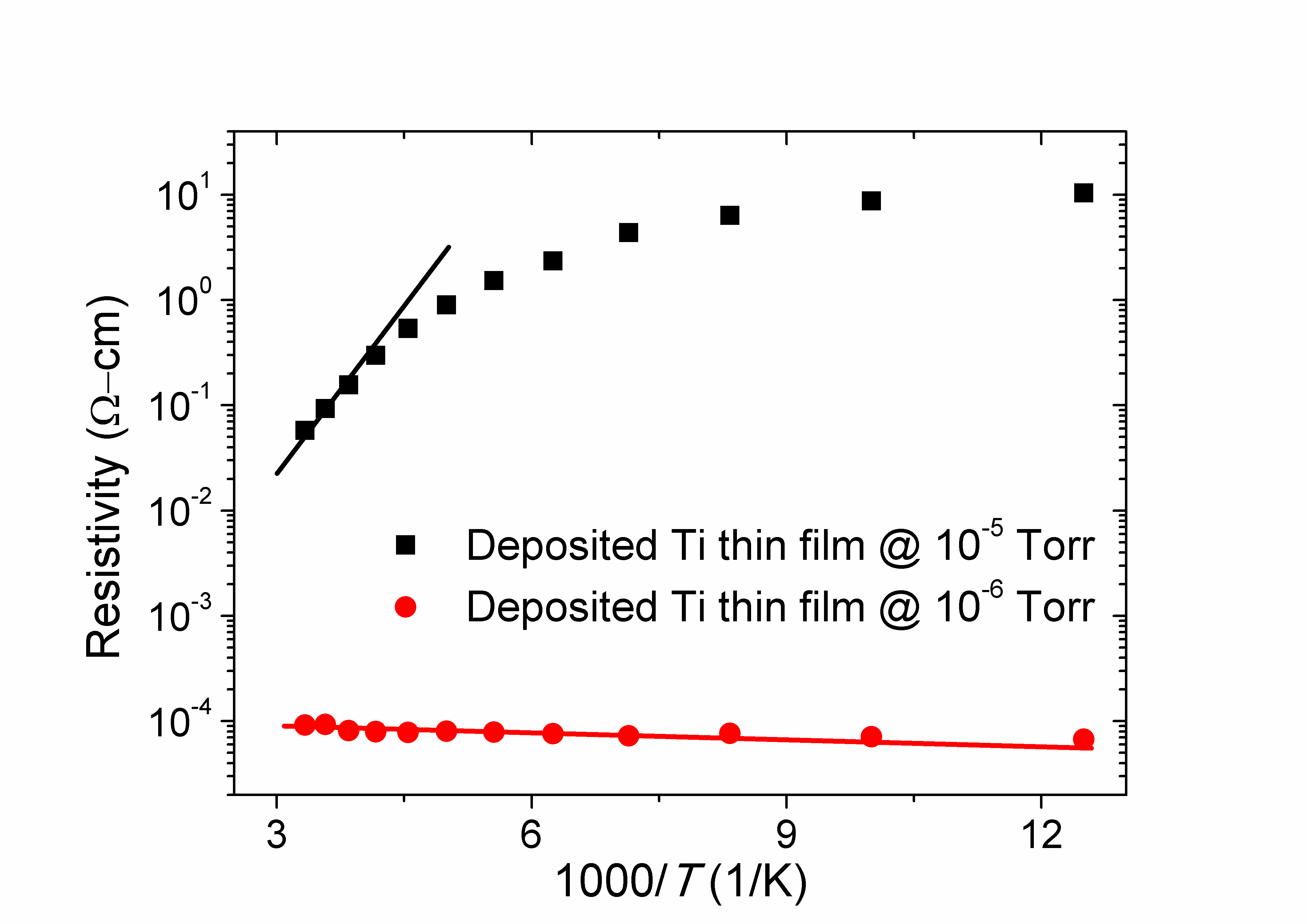
**

**Figure S6:** Resistivity as a function of inverse temperature of Ti thin films deposited under different base pressures (black square: ~10^-5^ Torr; red circle: ~10^-6^ Torr). The solid lines are guides for the eye.

1. **Responsivity and quantum efficiency of NW devices.**

An extensive description of the responsivity and an introduction of quantum efficiency of InP NW devices are given here. The responsivity, ***R***, is one of the important parameters in optoelectronic devices that decide the device performance with regard to the generated photocurrent ($\Delta I$) per incident optical power ($P_{opt}$) at a given wavelength,**^[S5]^**

***R*** $=\frac{\Delta I}{P_{opt}}$, (1)

where $P_{opt}$is defined as the light intensity multiplied by the effective illumination area. The unit of the responsivity is usually expressed as amperes per unit watt. In the main text, we use InP NW devices to examine the NC disorder effect on the photosensitivity. The excitation light was about 0.5 W/cm^2^. The effective illumination area is determined by the NW length (10^-4^ cm) multiplied by the NW diameter (2 × 10^-6^ cm) thus $P_{opt}$ is estimated to be about 10^-10^ W. The photocurrent variation, $\Delta I$, at applied voltage of 1 V are 10 and 0.2 nA for the NW- and NC- dominated devices which have RT resistances of 22 and 4600 MΩ, respectively. Hence the responsivity are 100 A/W and 2 A/W for the NW- and NC-dominated devices. It is worth noting that the responsivity of this NW-resistance dominated device is significantly larger, up to several times, than those reported previously.**^[S6-S8]^** In other words, these InP NWs, synthesized by self-seeded, solution-liquid-solid method, not only possess very low native point defects and low carrier concentration, but also reveal potentially promising optoelectronic responses. In addition, the quantum efficiency, *η*, is defined as the number of carrier generated per incident photo of the formula

$\eta=\frac{\frac{\Delta I}{q}}{\frac{P_{opt}}{h\nu}}$, (2)

where *q* and *h*ν are the charge and the incident photo energy. In this study, the green light laser having a wavelength of 532 nm was used and the incident power, *P_opt_*, was 10^-10^ W, yielding a flux of 2.68 × 10^8^ photons per second on the NW. The numbers of excitation carriers per second are 7 × 10^10^ and 10^10^ with quantum efficiencies of ~240 and ~37 for the NW- and NC-dominated devices. Map of the quantum efficiency as a function of applied voltage and the RT device resistance is shown in **Figure S7** for a reference.

**
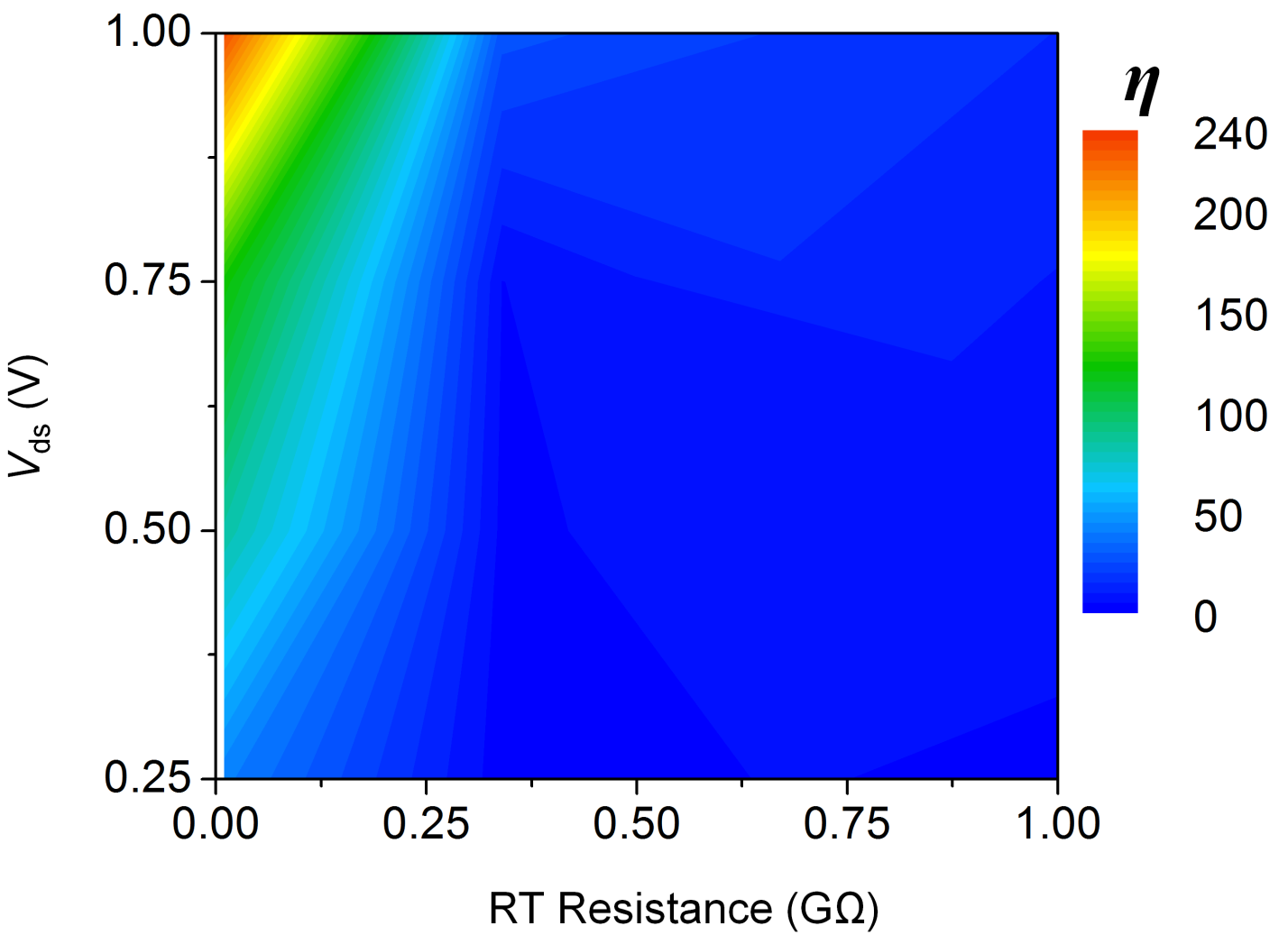
**

**Figure S7:** Map of quantum efficiency as a function of applied voltage and the RT device resistance.

**Reference**

[S1] Lin, Y. F.; Chen, T. H.; Chang, C. H.; Chang, Y. W.; Chiu, Y. C.; Hung, H. C.; Kai, J. J.; Liu, Z.; Fang, J.; Jian, W. B. *Phys. Chem. Chem. Phys.* **2010**, 12, 10928-10932.

[S2] Lin, Y. F.; Jian, W. B.; Wang, C. P.; Suen, Y. W.; Wu, Z. Y.; Chen, F. R.; Kai, J. J.; Lin, J. J. *Appl. Phys. Lett.* **2007**, 90, 223117.

[S3] Lin, Y. F.; Chiu, S. C.; Wang, S. T.; Fu, S. K.; Chen, C. H.; Xie, W. J.; Yang, S. H.; Hsu, C. S.; Chen, J. F.; Zhou, X.; Liu, Z.; Fang, J.; Jian, W. B. *Electrophoresis* **2012**, 33, 2475.

[S4] Berlincourt, T. G. *Phys. Rev.* **1959**, 114, 969.

[S5] Sze, S. M.; Ng, K. K. *Physics of Semiconductor Devices*; John Wiley & Sons: Edison, NY, **2007**; p666.

[S6] Wang, J.; Gudiksen, M. S.; Duan, X.; Cui, Y.; Lieber, C. M. *Science* **2001**, 293, 1455-1457.

[S7] Kind, H.; Yan, H.; Messer, B.; Law, M.; Yang, P. *Adv. Mater.* **2002**, 14, 158.

[S8] O’Brien, G. A.; Quinn, A. J.; Tanner, D. A.; Redmond, G. *Adv. Mater.* **2006**, 18, 2379.
